# The Effect of Laser Irradiation to Surfaces of Computer-Aided Design/Computer-Aided Fabrication Resin Blocks Coated with a Silane Coupling Agent on Bond Strength between the Resin Blocks and Composite Resin

**DOI:** 10.3390/dj11120290

**Published:** 2023-12-13

**Authors:** Hiroshi Ohno, Masaya Suzuki, Koichi Shinkai

**Affiliations:** 1 Advanced Operative Dentistry, Graduate School of Life Dentistry at Niigata, The Nippon Dental University, Niigata 951-8580, Japan; ohno109@ngt.ndu.ac.jp; 2 Department of Operative Dentistry, School of Life Dentistry at Niigata, The Nippon Dental University, Niigata 951-8580, Japan; collagen@ngt.ndu.ac.jp

**Keywords:** CAD/CAM resin blocks, silane heat treatment, shear bond strength, semiconductor laser

## Abstract

The aim of this study was to investigate the effect of laser irradiation to computer-aided design/computer-aided fabrication (CAD/CAM) resin blocks coated with a silane coupling agent on the bond strength between resin blocks and composite resin. The CAD/CAM resin blocks used in this study were Cerasmart 300 (GC) and Vita Enamic (Vita); they were cut into plates and then subjected to a series of treatments. After processing with a silane coupling agent, treatment with a semiconductor laser was performed at 3.0, 5.0, and 7.0 W, followed by bonding procedures. The control group included those exposed to silane and bonded without laser application. After bonding, a mold with a simulated cavity was formed on the specimen and filled with flowable composite resin, and they were stored for 24 h or stressed by thermal cycling for subsequent testing that assessed the shear bond strength (n = 10). The results revealed that the bond strength was significantly enhanced by laser irradiation after applying a silane coupling agent (*p* < 0.03), whereas significant increase was not detected between the materials (*p* > 0.05). Particularly, 7 W laser irradiation had a significant increase on the bond strength between the composite resin and Cerasmart block after thermal cycling (*p* = 0.009). The SBS of the composite resin to CAD/CAM resin blocks was significantly enhanced by laser irradiation after silane coupling agent application.

## 1. Introduction

Recently, crown and inlay restorations using computer-aided designed/computer-aided manufactured (CAD/CAM) resin blocks have been employed in clinical practice [1,2,3]. CAD/CAM-generated composite resin inlays provide better reproducibility and reduce production costs through standardized manufacturing processes [4]. Several CAD/CAM composite resin blocks are available in the market, such as Vita Enamic (VITA Zahnfabrik, Bad Säckingen, Germany), Lava Ultimate (3M ESPE, North Cordova, IL, USA), and Cerasmart (GC Corporation, Tokyo, Japan). These composite blocks were produced by means of either conventional technology with nanotechnology or polymer-infiltrated ceramic network (PICN) technology [4]. Compared with glass-ceramics/ceramics, which are the most familiar CAD/CAM materials, CAD/CAM composites resin blocks show better machinability and higher marginal adaptation due to their lower brittleness [5]. However, CAD/CAM-generated composite resin restorations may result in marginal fracture after long-term clinical performance due to lower fracture toughness [5,6]. Restorations fabricated with CAD/CAM resin blocks may be damaged by an excessive load in occlusal or other directions [6,7]. Depending on the degree of damage, a composite resin repair is performed to reduce the number of treatments and diminish the burden associated with laboratory procedures. To obtain a good long-term outcome, the composite resin should form a strong bond with the CAD/CAM composite resin block. Sandblasting and hydrofluoric acid treatment are the main procedures for this purpose [8]. Although the former is used in the laboratory and yields excellent outcomes in improving bond strength, the treatment at some restoration sites may be difficult in a clinical setting, because the powder used in the former may be scattered in the oral cavity, causing patient discomfort. Furthermore, adhesive treatment with hydrofluoric acid is contraindicated in the oral cavity [9].

Several studies have examined the surface treatment of CAD/CAM materials considering the properties of lasers; however, most of them have focused on utilizing surface roughness caused by a high absorption of water in the material surface with subsequent micro-explosion using high-power lasers such as Er:YAG and CO_2_ [10,11,12,13,14]. Exothermic action is also one of the main properties of lasers; however, few previous studies have investigated it for the surface treatment of CAD/CAM materials. Therefore, we focused on the activation of silane coupling agents by heat treatment resulting from laser irradiation to obtain sufficient bond strength between CAD/CAM resin blocks and composite resins.

The methoxy groups of the silane coupling agent ɣ-MPTS are hydrolyzed during silane coupling, forming silanol groups, which bond to siloxane groups on the filler’s surface in the composite resin, and the chemical bond of ɣ-MPTS to a resin monomer with methacryloyl groups is formed [15]. Heating ɣ-MPTS is effective in accelerating this series of reactions. Studies have reported that silane coupling agents activated by heating improve bond strength, whereas the values of heating temperatures required for this are unclear, and heating methods using dryers or ovens are not applicable in the oral cavity [16,17]. Conversely, heating treatment derived from laser irradiation can be done in the oral cavity. Although several studies have assessed the effect of the heating treatment of silane coupling agents with lasers on the bond strength between various restorative materials and composite resin in the laboratory [18,19,20,21], points are ambiguous regarding the type of laser, output power, or heating temperature.

Recently, the CAD/CAM method has been applied to the fabrication of zirconia crowns and dentures [22,23]. Although, various methods have been investigated to improve the bonding between zirconia and resin cements, but a reliable bonding method has not yet been established [22]. Further dentures milled from resin blocks by the CAD/CAM method may require special bonding treatment to the fracture surface in case of fracture repair because the composition of the PMMA resin in the block is different from that of conventional dentures [23]. The results of this study may be useful in such cases.

Thus, this study aimed to evaluate the efficacy of the heat treatment of silane coupling agents by laser irradiation in enhancing the bond strength between resin blocks and composite resins. The null hypothesis is that heating of the silane coupling agent using a semiconductor laser does not influence the bond strength between CAD/CAM resin blocks and composite resin.

## 2. Materials and Methods

### 2.1. Experimental Materials

The materials used in this experiment are shown in Table 1. In this study, two types of CAD/CAM blocks, namely, hybrid resin block, Cerasmart 300 (CS, GC, Tokyo, Japan), and hybrid ceramic (polymer infiltrated ceramic network) block, Vita Enamic (EN, Vita Zahnfabrik, Bad Sackingen, Germany), were used. Clearfil Majesty ES Flow (Kuraray Noritake Dental, Tokyo, Japan), Clearfil Ceramic Primer Plus (Kuraray Noritake Dental), and Clearfil Universal Bond Quick ER (Kuraray Noritake Dental) were selected as a flowable composite resin, silane coupling agent, and bonding agent, respectively.

### 2.2. Plate Preparation

The CAD/CAM resin block was divided into 3.0 mm thick plates with a low-speed precision cutting machine (Isomet Low Speed, Buehler, Lake Bluff, IL, USA). The surfaces of the prepared plates were wet polished with #600 emery paper for 1 min under water irrigation and then ultrasonically cleaned for 5 min to provide the adherend surface. The total number of samples in the plate was 120.

### 2.3. Specimen Preparation

The adhesive treatments for each experimental group are summarized in Table 2. Using G*power software (version 3.1.9.7; Franz Faul University, Kiel, Germany), a power analysis was conducted with an effect size of 0.4 (Cohen’s large effect size) and a power of 0.8, resulting in a sample size of 73. Since the total number of experimental groups was 8, the required number of samples per group was calculated to be 9.125; thus, the number of samples for each group was 10.

Control group: After applying Clearfil Ceramic Primer Plus (Kuraray Noritake Dental) to the surface of a plate using a micro-brush, medium-pressure air blowing was immediately performed for 3 s. A stopper was attached to the air lever of the three-way syringe to ensure that a medium air pressure was always achieved. Then, the plate was treated with the Clearfil Universal Bond Quick ER (Kuraray Noritake Dental), followed by medium-pressure air blowing for 10 s to ensure the ceased movement of the liquid surface, which was then followed by light irradiation for 5 s at 2.0 W using a light-curing unit (Pencure 2000, Morita, Tokyo, Japan) with 2000 mW output (n = 10).

Laser irradiation group: After applying ceramic primer and air blowing, as mentioned above, the plate surface was exposed to semiconductor laser irradiation (wavelength 810 nm, P2 Dental Laser System, Pioon Laser Technology Inc., Wuhan, China), and the irradiation method was as follows. The laser handpiece with an irradiation tip (8.0 mm in diameter) was mounted on a flexible arm of a fixed table. The plate surface applied with the silane coupling agent was irradiated at 3.0, 5.0, and 7.0 W for 60 s. The distance between the plate surface and the irradiation tip, denoted as the laser irradiation distance, was 1.0 mm. The subsequent bonding procedure was the same as in the control group. The experimental groups with CS and 3.0, 5.0, and 7.0 W laser irradiation were coded as CSC, CS3, CS5, and CS7, respectively, and those with EN and 3.0, 5.0, and 7.0 W laser irradiation were labeled ENC, EN3, EN5, and EN7, respectively (n = 10).

A cylindrically simulated cavity was formed within a mold (diameter, 2.0 mm; height, 3.0 mm) that was fabricated with a silicone rubber impression material (Examix Fine Injection Type, GC). The obtained silicone mold was positioned and fixed in the center of the block surface and subjected to each adhesive method. The simulated cavity was filled with two layers of flowable resin in layers of 1.5 mm each and photocured for 10 s each with Pencure 2000 (Morita) with 2000 mW output, followed by the removal of the silicon mold.

### 2.4. Shear Bond Strength (SBS) Test

Adhesive specimens were preserved in a constant temperature and humidity chamber (37.0 °C, 95% relative humidity) for 24 h before the SBS test. A specimen was placed in the center of a specimen fixation ring so that the adhesive surface of the specimen protruded slightly from it and was parallel to the edge of the ring made from autopolymerizing resin. After confirming that the resin used for fixation was sufficiently cured after more than 30 min, SBS tests were done with a tabletop testing machine (EZ Test 500N, Shimadzu, Kyoto, Japan) at a crosshead speed of 1.0 mm/min.

The obtained data were statistically analyzed by the two-way analysis of variance (ANOVA), with the material type and laser irradiation power as main factors, followed by the Tukey post hoc test to compare the values of the studied parameter among the experimental groups (α = 0.05). The Bell Curve^®^ for Excel version 3.20 (Social Survey Research Information, Tokyo, Japan) was employed for descriptive statistics.

**Figure 1:**
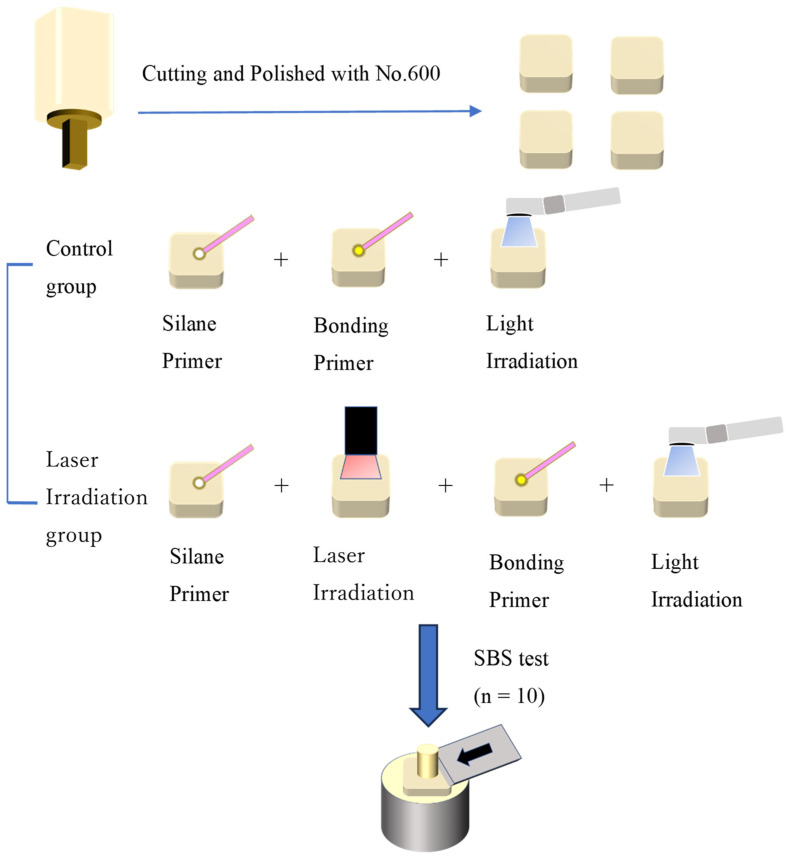
Flowchart from specimen preparation to shear adhesion test.

The flow from specimen preparation to shear adhesion testing is schematically shown in Figure 1.

### 2.5. SBS Test after the Thermal Cycling Load Test

The irradiation output with the highest adhesion strength was selected from the outcomes of the first SBS test and was used to prepare adhesive specimens for the SBS test after the thermal cycling load test. The CS and EN in the laser-treated group were obtained using the selected irradiation power, and those for the control group were also received (n = 10). Thermal cycling load tests were conducted on them using a long-term durability tester (Thermal Cycling K178, Tokyo Giken). One thermal cycle consisted of 5 °C for 30 s, transfer for 10 s, 55 °C for 30 s, and another for 10 s. These processes were repeated 10,000 times within 10 days. After the thermal cycling load test, the SBS test was conducted as described above. Moreover, t- or Mann–Whitney U tests were used for the analysis of variables according to the equal variances for each material (α = 0.05). Descriptive statistics were outlined using Bell Curve^®^ for Excel version 3.20 (Social Survey Research Information, Tokyo, Japan).

### 2.6. Failure Mode Analysis

Failure modes after SBS test were evaluated using a stereomicroscope (SZX7, Olympus) at 20×. Failure modes were defined as adhesive failure (AF; failure restricted within the adhesive area), mixed failure (MF; failure extending from the adhesive to either the composite resin or plate area), and cohesive failure (CF, failure limited by the composite resin or plate area). 

### 2.7. Scanning Electron Microscopy (SEM) Observation

Some representative specimens were chosen from each of the experimental groups to observe the micromorphology of adhesive surfaces after SBS test. After sputter-coating the fractured surfaces of the plate sites with palladium and platinum, they were assessed via SEM (TM4000Plus Miniscope, Hitachi, Tokyo, Japan) at a voltage of 5 kV.

### 2.8. Measurement of Plate Surface Temperatures after Laser Irradiation

Each surface of the plates was subjected to semiconductor laser irradiation at 3.0, 5.0, and 7.0 W, and the surface temperature of each plate was measured immediately thereafter with an infrared radiation thermometer (Testo 830-T2, Azwan, Osaka, Japan). The laser irradiation time and distance were 60 s and 1.0 mm, respectively. The measurements were observed 10 times, and the average value was introduced as the plate surface temperature at each irradiation power. Given the equal distribution of data by Rubin’s test, a two-way ANOVA was performed (α = 0.05).

## 3. Results

### 3.1. SBS

**Figure 2:**
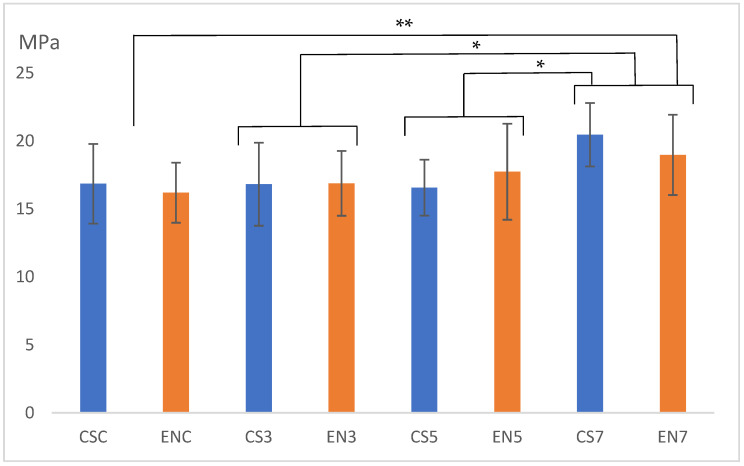
Shear bond strength values after 24 h of storage. * *p* < 0.05, ** *p* < 0.01.

The SBS values of the studied groups after 24 h of storage are demonstrated in Figure 2. The mean (SD) of the bond strength (MPa) of each experimental group was as follows: CSC, 16.8 (2.92); CS3, 16.8 (3.06); CS5, 16.6 (2.05); CS7, 20.5 (2.33); ENC, 16.2 (2.21); EN3, 16.9 (2.38); EN5, 17.7 (3.53); and EN7, 19.0 (2.95).

Owing to the equal distribution of data proved by Rubin’s test, a two-way ANOVA was performed. The results of the two-way ANOVA are shown in Table 3. The types of materials used did not differ significantly (*p* = 0.723), whereas an opposite trend was observed for laser power (*p* = 0.003). No interaction effect was found between the two factors indicated (*p* = 0.514). Therefore, further statistical analysis was carried out only in the laser power subgroups. The results showed significant differences between the 7 W and control (without laser irradiation group) (*p* = 0.004), 3 W (*p* = 0.012), and 5 W (*p* = 0.029) groups; however, no significant difference was detected among control, the 3 W groups, and the 5 W groups (*p* > 0.723). Therefore, the results of this statistical analysis imply that the bond between the composite resin and the CAD/CAM hybrid resin blocks was significantly strengthened by 7 W laser irradiation to ceramic primer-applied surfaces; however, the effect did not significantly differ between the two CAD/CAM blocks used in this study.

**Figure 3:**
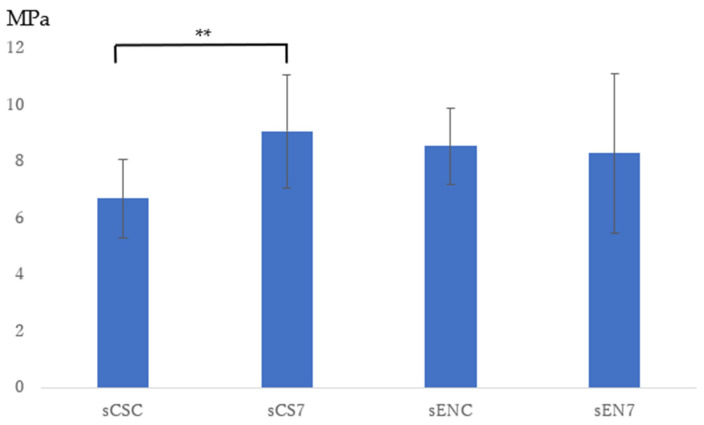
Shear bond strength after the thermal cycle loading test. ** *p* < 0.01.

The highest average SBS values after 24 h of storage were noted for both CS and EN (CS7 and EN7) in the 7 W group. Hence, specimens for the groups that received this dose and the control group were newly prepared to investigate bond strength after being subjected to thermal cycle stress. According to the material, the resulting four experimental groups were coded as sCSC, sCN7, sENC, and sEN7 after loading thermal cyclic stress. The SBS values of the experimental groups that were identified after the test are reflected in Figure 3. The mean (SD) of the bond strength (MPa) of each experimental group was as follows: sCSC, 6.69 (1.39); sCS7, 9.07 (1.99); sENC, 8.55 (1.36); and sEN7, 8.29 (2.82). The results of the t-test for the comparison of sCSC and sCS7, revealing equal variances, also demonstrated significant differences between them (*p* = 0.009). Meanwhile, the results of the Mann–Whitney U test for the comparison of values between sENC and sEN7, which did not show equal variances, demonstrated no significant differences between them (*p* = 0.853). 

### 3.2. Results of the Analysis of Failure Modes

Figure 3 shows the failure modes of the samples that were subjected to SBS tests after 24 h of storage. The failure modes of all CS groups were judged as AF. The failure modes of ENC, EN3, EN5, and EN7 were judged as 20% AF + 60% MF + 20% CF, 70% AF + 30% MF, and 50% AF + 50% MF, respectively. In addition, the failure modes of all specimens subjected to loaded thermal cycle stresses were judged as AF.

### 3.3. SEM Images of Specimens Representing Each Failure Mode

**Figure 4:**
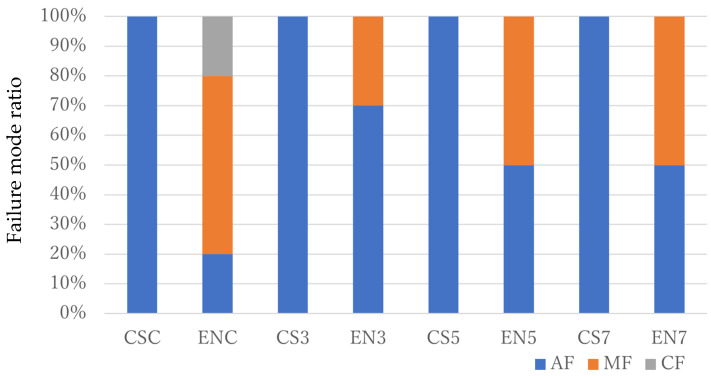
Results of the failure mode analysis of specimens after 24 h of storage.

SEM images of representative specimens for AF, MF, and CF are displayed in Figure 4.

**Figure 5:**
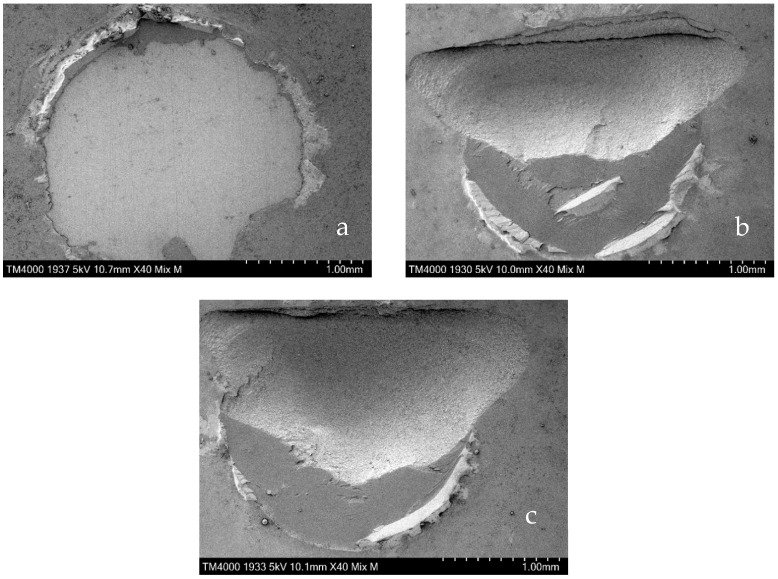
SEM images of representative specimens judged as AF (**a**), MF (**b**), and CF (**c**).

On the SEM image of the specimen identified as AF, adhesion fractures nearly occurred at the adhesive interface between the block and the composite resin (Figure 5a. The SEM image of the specimen judged as MF demonstrates approximately 50% of the bond surface covered by adhesion fractures between the block and the composite resin and 50% of the block surface containing cohesive fracture (Figure 5b). On the other SEM image of the specimen marked as CF, CF in the block was found on approximately 70% of the total adhesion surface (Figure 5c).

### 3.4. Surface Temperature of the Plates Estimated Immediately after Laser Irradiation

**Figure 6:**
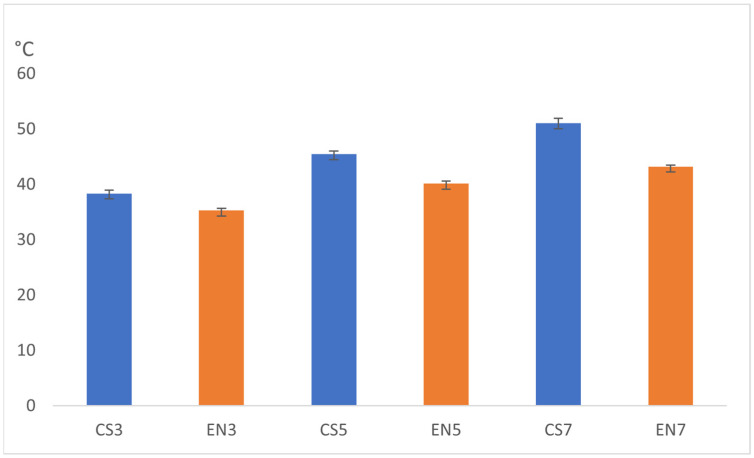
Surface temperature of the plates estimated immediately after laser irradiation.

The mean (SD) of the plate surface temperature (°C) immediately after laser irradiation for CS3, CS5, CS7, EN3, EN5, and EN7 were 38.4 (0.6), 45.5 (0.6), 51.1 (0.9), 35.3 (0.4), 40.1 (0.5), and 43.2 (0.3), respectively. The surface temperature of the control group (CSC and ENC) was 29.0 °C. (Figure 6.)

Given the equal distribution of data by Rubin’s test, a two-way ANOVA was performed, and the results are summarized in Table 4. Because significant differences were detected in the material type, laser power, and interaction between these factors (*p* < 0.001), a simple main effect test was performed to estimate the role of each factor. The outcomes showed a significant difference between CS and EN at all laser powers (*p* < 0.001), with CS exhibiting significantly higher surface temperature than EN immediately after laser irradiation. Significant differences were also identified among the laser powers for both materials (*p* < 0.001), showing a positive correlation between higher power and higher surface temperatures immediately after laser irradiation.

## 4. Discussion

The main objective of this study was to determine the effect of heat treatment of silane coupling agents by semiconductor laser irradiation on the bond strength between CAD/CAM resin blocks and composite resins. Statistical analysis revealed that heat treatment by 7 W laser irradiation to the resin block surface coated with the silane coupling agent significantly improved the bond strength between the CAD/CAM resin block and the composite resin. Therefore, the null hypothesis that heat treatment of silane coupling agents with a semiconductor laser does not affect the bond strength between CAD/CAM resin blocks and composite resin was rejected.

Heat treatment by laser irradiation can remove alcohol, water, and other by-products from a resin block surface with a silane coupling agent applied; however, it has been reported to accelerate the processes in silane condensation [15,18,19,20], where the formation of siloxane bonds is promoted, thus elevating bond strength. The semiconductor laser used in this study appeared to be suitable for the heat treatment of the resin block applied with a silane coupling agent, because its application did not deteriorate the structure of the resin block surface and hindered the evaporation of the silane coupling agent, even when the laser power was increased. On the contrary, the processing of surfaces with Er:YAG or CO_2_ lasers can induce minute cracks on the resin block surface at the time of heating, depending on the output power [10,13]. In addition, the silane coupling agent applied on the resin block surface may evaporate when irradiated with an Er:YAG or CO_2_ laser, because these types are characterized by a high absorption of water, which is a component of silane coupling agents. Hence, irradiation by Er:YAG or CO_2_ lasers is unsuitable for the heat treatment of resin blocks treated with silane coupling agents.

From the findings of this study, only the CS7 group that was subjected to irradiation showed significantly higher bond strength than the control group, and the mean temperature of the surfaces of the CS7 specimens was 51.1 °C after 1 min of treatment. Therefore, heat treatment at more than 50 °C may be necessary to activate silane coupling agents. A previous study reported that heat treatment at approximately 50 °C was equally or even more effective in activating silane coupling agents than that of 100 °C [15,21]. This report overall supports the results of this study. Another study reported that heat treatment at approximately 38 °C was effective in activating silane coupling agents [16]. However, this study demonstrated that high bond strength did not result from heat treatment at approximately 38 °C.

Among blocks commonly utilized for CAD/CAM, CS and EN are classified as hybrid resins in terms of material type. However, their structures are different. That is, EN has a structure of polymer infiltrated in a ceramic network, whereas CS has that of glass nanofillers scattered in a matrix resin, like a composite resin for dental restorations. The amount of inorganic material supposed to be involved in silane coupling treatment is higher in EN (86%) than in CS (71%). Thus, the bond-enhancing effect of silane coupling treatment on EN was speculated to be stronger than that on CS, whereas the findings highlighted no statistically significant difference between the two materials for all laser irradiation conditions. This may suggest that the bond strength between the matrix resin in the block and the composite resin monomer is higher than that between the filler in the former and the latter. On the contrary, the heating effect yielded following laser irradiation was higher for CS than for EN. This is presumably because the increase in the surface temperature caused by laser irradiation was more pronounced for CS than for EN, and the silane coupling agent was more active because of heating.

The experimental groups of both CS and EN, whose silane coupling agent-treated surfaces were irradiated with 7 W power, exhibited the highest bond strength after 24 h of storage; hence, thermal cycle loading tests were conducted on these experimental groups and the control group. The results of this experiment revealed that EN showed no significant difference in bond strength between the study and control group, whereas that of the CS group differed significantly. The adhesive strength of the specimens subjected to thermal cycling for both CS and EN was significantly lower than that of the specimens after 24 h without thermal cycling. This may be due to the difference in the thermal expansion coefficient between the resin block and the composite resin. In other words, it is presumed that the bond strength may have decreased due to stress loading on the adhesive interface caused by the thermal cycle test and hydrolysis of the silane coupling agent [24]. Laser irradiation at 7 W output increased the surface temperature of CS to over 50 °C, whereas the surface temperature of EN remained at 43 °C. As a result, the activation whereas of the silane coupling agent by the heat of laser irradiation was significant in the CS, the activation was probably less pronounced in the EN. We speculate that the 7 W laser irradiation activated the silane coupling agent applied to the CS with the irradiation heat, and strengthened the hydrogen bonding via silanol and hydroxyl groups, thus resulting in a smaller decrease in the adhesive strength of the CS after the thermal cycle test compared to EN. This result suggests that the heat treatment of silane coupling agents applied at more than 50 °C to block surfaces can potentially improve the long-term adhesive durability of CAD/CAM resin blocks and composite resin.

The most concerning adverse event of semiconductor laser irradiation is heat generation in dental pulp, and several studies have outlined this complication [25,26,27]. These studies have revealed that pulp necrosis can occur when the temperature in the pulp chamber rises above 5.5 °C following laser irradiation, whereas temperature increases between 3.3 °C and 5.5 °C are considered to cause only reversible inflammation. In a previous study, a semiconductor laser with a wavelength of 809 nm and a beam diameter of 0.6 mm was used to irradiate extracted third molars at an output power of 7 W, irradiation distance of 5 mm, irradiation time of 15 s, pulse of 16 Hz, and energy density of 74 J/cm^2^ to estimate the temperature in the pulp cavity. As a result, the observed temperature increase was 5.25 ± 0.55 °C [28]. Therefore, it is possible to irradiate the pulp with a laser power as high as 7 W without affecting the pulp by properly adjusting the irradiation time, distance, and irradiation mode. In addition, studies that have measured pulp cavity temperatures in response to such laser irradiation have mainly used untreated human extracted and bovine teeth [27,28,29,30]. However, when conducting dental restoration in vivo, a laser beam penetrates resinous or ceramic materials, as well as the tooth substance, until it reaches the pulp. Speculatively, the heat effects of laser irradiation may be less likely to reach the pulp with lasers used in dental restorations because resinous and ceramic materials are less thermally conductive than the tooth substance.

Previous studies have reported that when CS and EN are treated with silane coupling agents after sandblasting, the SBS of the composite resin to CS and EN were approximately 34 and 38 MPa, respectively. [31,32,33,34]. In this study, when blocks applied with a silane coupling agent were irradiated using a semiconductor laser with a power output of 7 W, the SBS was approximately 20 MPa for CS7 and 19 MPa for EN7. Compared with the bond strength reported in these studies, ours were approximately one-half to two-thirds; however, the bond strength of approximately 20 MPa appears clinically sufficient for the bond strength of the composite resin used in the repair of CAD/CAM restorations [35,36]. Moreover, sandblasting is not practical in the oral cavity, because the complete suctioning of the flying alumina oxide powder is challenging. Therefore, as an alternative to sandblasting, semiconductor laser irradiation after silane coupling agent application is recommended as an effective treatment for enhancing the bond strength between CAD/CAM materials and composite resin when repairing CAD/CAM restoration. Furthermore, within laboratory settings, the bond strength of CAD/CAM crowns may be increased by irradiating them with a semiconductor laser after sandblasting and silane coupling treatment.

In this study, only composite resin-based CAD/CAM blocks were subjected to bonding tests. In the future, other CAD/CAM blocks such as zirconia and lithium disilicate glass ceramics should also be evaluated. Moreover, examining the bond strength using other silane coupling agents and CAD/CAM resin blocks with various compositions may be necessary. Further studies using semiconductor lasers with different wavelengths are needed to estimate the optimal laser irradiation parameters for the heat treatment of silane coupling agents.

## 5. Conclusions

The bond strength between the composite resin and CAD/CAM resin block was significantly improved when the CAD/CAM resin block surface was coated with a silane coupling agent followed by laser irradiation at 7 W. Moreover laser irradiation at 7 W maintained or improved the bond strength between the composite resin and CAD/CAM resin block, even after thermal cycling.

## Figures and Tables

**Table 1:** Experimental materials used in this study.

Material	Code	Lot#No.	Composition	Manufacturer
Cerasmart 300	CS	2302206	Bis-MEPP, UDMA, DMA (29 wt%), SiO_2_ and B_2_O_3_ glass nanofillers (71 wt%)	GC
Vita Enamic	EN	97110	Bis-GMA, UDMA, Bis-EMA, TEGDMA, polymer network (14 wt%), SiO_2_, Al_2_O_3_, Na_2_O, K_2_O, B_2_O_3_, ZrO_2_, CaO ceramic network (86 wt%)	Vita
Clearfil Majesty ES Flow	–	A30347	Surface-treated barium glass, surface-treated silica fillers, monomer (TEGDMA and methacrylic acid monomer), photopolymerization catalyst, stabilizing agent, coloring agent	Kuraray Noritake Dental
Clearfil Ceramic Primer Plus	–	B10087	Silane coupling agents, monomer (MDP), and ethanol	Kuraray Noritake Dental
Clearfil Universal Bond Quick ER	–	4J0349	Monomer (Bis-GMA, phosphate ester monomer: MDP, HEMA, hydrophilic amide monomer), filler (silica-based micro filler), ethanol, photopolymerization catalyst, scientific polymerization accelerators, purified water, and NaF	Kuraray Noritake Dental

Bis-MEPP: 2, 2’-bis (4-methacryloxy polyethoxyphenyl) propane, UDMA: urethane dimethacrylate, DMA: dimethacrylate, Bis-GMA: bisphenol-A-glycidylmethacrylate, Bis-EMA: bisphenol-A polyethylenglycol dietherdimethacrylate, TEGDMA: triethylene glycol dimethacrylate, MDP: 10-methacryloxydecyldihydrogenphosphate, HEMA: 2-hydroxyethyl methacrylate.

**Table 2:** Experimental group codes.

Group Code	Laser Irradiation Power	n	Thermal Cycling Load Test
CSC	without irradiation	10	Without loading
CS3	3 W		
CS5	5 W		
CS7	7 W		
ENC	without irradiation		
EN3	3 W		
EN5	5 W		
EN7	7 W		
sCSC	without irradiation	10	With loading of 10,000 cycles
sCS7	7 W		
sENC	without irradiation		
sEN7	7 W		

**Table 3:** Results of the two-way analysis of variance of shear bond strength values in the groups after 24 h of storage.

Factor	Type III Sum of Squares	Degrees of Freedom	Mean Square	F Value	*p* Value
Material Type	1.044	1	1.04	0.12	0.72
Laser Power	128.44	3	42.81	5.19	0.003
Material Type * Laser Power	19.04	3	6.34	0.77	0.51
Error	592.91	72	8.23		
Overall	741.44	79			

**Table 4:** Results of the two-way ANOVA test of block surface temperatures assessed immediately after laser irradiation.

Factor	Type III Sum of Squares	Degrees of Freedom	Mean Square	F Value	*p* Value
Material Type	440.10	1	440.10	1279.44	<0.001
Laser Power	1069.43	2	534.71	1554.49	<0.001
Material Type * Laser Power	57.63	2	28.81	83.77	<0.001
Error	18.57	54	0.34		
Overall	1585.74	59			

## Data Availability

The data presented in this study are available on request from the corresponding author.
